# Chemoinformatics and structural bioinformatics in OCaml

**DOI:** 10.1186/s13321-019-0332-0

**Published:** 2019-02-05

**Authors:** Francois Berenger, Kam Y. J. Zhang, Yoshihiro Yamanishi

**Affiliations:** 1 Department of Bioscience and Bioinformatics, Faculty of Computer Science and Systems Engineering, Kyushu Institute of Technology, Iizuka, Fukuoka Japan; 2 Laboratory for Structural Bioinformatics, Center for Biosystems Dynamics Research, RIKEN, Yokohama, Kanagawa Japan; 3 PRESTO, Japan Science and Technology Agency, Kawaguchi, Saitama Japan

**Keywords:** Chemoinformatics, Structural bioinformatics, Bisector tree, Scientific software, Software prototyping, Open source, Functional programming, OCaml

## Abstract

**Background:**

OCaml is a functional programming language with strong static types, Hindley–Milner type inference and garbage collection. In this article, we share our experience in prototyping chemoinformatics and structural bioinformatics software in OCaml.

**Results:**

First, we introduce the language, list entry points for chemoinformaticians who would be interested in OCaml and give code examples. Then, we list some scientific open source software written in OCaml. We also present recent open source libraries useful in chemoinformatics. The parallelization of OCaml programs and their performance is also shown. Finally, tools and methods useful when prototyping scientific software in OCaml are given.

**Conclusions:**

In our experience, OCaml is a programming language of choice for method development in chemoinformatics and structural bioinformatics.

## Introduction

There are several schools of thought in computer programming. Each school is represented by several programming languages and some languages are multi-paradigm.

In declarative languages (like SQL), a programmer writes a kind of mathematical specification of what to compute, and the compiler will automatically derive a program implementing this specification. Prolog [1], is also such a programming language where the specification is given as a collection of logic predicates.

On the contrary, in imperative programming, the programmer writes in extensive details how to compute the result he wants. Ada, C, Fortran and Pascal are famous representatives of this style of programming.

In Object-Oriented programming, data structures and the allowed operations on them are grouped into classes. Classes can be hierarchically organized, and behavior inherited so that generic code can be reused between software components. C++, Java, Eiffel, Ruby and Python are famous members of this family of languages. Most Object-Oriented languages use the imperative style of programming.

In functional programming, a program is a collection of functions. State passing is done explicitly via function parameters. Functional programming has a mathematical taste and dates back to Lisp. Lisp, Scheme, OCaml, F#, Haskell, Scala, Racket and Clojure are representatives of the functional style of programming. There are several advantages to using functional programming [2]. Since state passing is explicit, functional programs are easy to reason about. They easily fit in the head of the programmer. Some functional programming languages are pure (e.g. Haskell); they guarantee referential transparency, the fact that an expression can be replaced by its corresponding value without changing the program behavior. There are some articles about the productivity boost associated with functional programming [3, 4].

While there are not many, some functional programming libraries for chemoinformatics do exist. In Haskell, the ‘smiles’ library [5] provides full support for the OpenSMILES specification [6]. While the ‘radium’ library [7] provides the periodic table plus readers and writers for SMILES and condensed formulas. In Scala, the ‘chem$^{\mathrm {f}}$’ library [8] provides a purely functional cheminformatics toolkit [9].

In this article, we concentrate on Objective Caml (OCaml [10]), in the context of scientific software prototyping for chemoinformatics and structural bioinformatics. OCaml is a general purpose functional programming language developed at INRIA, the French national research institute for computer science, robotics and applied mathematics. OCaml focuses on expressiveness and safety. Some of the language’s strengths include its type system, with parametric polymorphism (called generics in Java, templates in C++) and type inference. Thanks to Hindley–Milner type inference [11, 12], the OCaml programmer is freed from explicitly providing function parameters and result types. For a course on programming languages and types, we refer interested readers to Pierce [13]. OCaml supports user-defined algebraic data types, records, sums/enums and pattern matching. Pattern matching is a generalization of the switch statement present in other languages. When pattern matching, a program is driven by the type of the parameter being matched upon. In OCaml, memory is managed automatically, by an incremental garbage collector, preventing memory corruption. Interactive use of OCaml is possible via a read-eval-print loop called the OCaml interpreter. Interacting with the interpreter is a standard way to test a function or to check that some functionality provided by a library works the way one understands it. In addition to its byte-code compiler and interpreter, OCaml offers a compiler that produces efficient executables. Tail-recursive functions are automatically translated to efficient loops by the OCaml compiler. OCaml also features an object-oriented layer, with multiple inheritance, parametric and virtual classes. While OCaml was initially used to develop symbolic computing applications, such as automatic theorem provers, compilers, interpreters and static program analyzers, it is now used to develop software in many other areas.

Functions are first-class values in OCaml. A function can be passed as an argument to, or returned by, another function. OCaml is a multi-paradigm language. For performance reasons, OCaml offers many imperative features (exceptions, modifiable variables, records, arrays and loop statements). OCaml built-in data types include not only integers, floating point numbers, booleans, characters and strings but also more advanced data types such as tuples, records, arrays and lists.

Large programs are easy to structure due to modules, which share some traits with classes in object-oriented programming. Modules can be organized hierarchically and parameterized over a number of other modules. Such a function, from modules to module is called a functor and allows high level generic programming.

OCaml’s evaluation strategy is strict. All parameters to a function are evaluated prior to entering the function’s body. The compilation of OCaml programs is fast. For example, the $\sim 3000$ OCaml lines (without comments) of the consent software [14] and its four executables compile from scratch and link in $\sim 3.8$ s (resp $\sim 1.1$) using dune (version 1.6.2) and a single core (resp. up to all cores) of our desktop computer (16 cores, Intel Xeon 2.1GHz, 64GB RAM, Linux Ubuntu 18.04.1 LTS).

Strong static types are types which are enforced by the compiler. Due to the use of types and garbage collection, several run-time errors which plague other programming languages are absent from OCaml programs: null pointer exception, dereference after free, type cast exception, segmentation fault, unhandled switch cases and most memory leaks. In functional programming, more complex properties can be encoded and statically enforced by structuring code using monads [15], which are pervasive in Haskell [16], or by using dependent types (not available in OCaml, but in Coq [17], Idris [18, 19] and Agda [20]). A function that is guaranteed to produce a result in a finite time is called total. Functions for which there is no such guarantee are called partial. For some functions, Idris can check if they are total. However, such advanced functional programming concepts are out of the scope of this article.

Despite not being very popular, OCaml is not a niche language. Most of its academic users work in computer science, on compilers and formal methods. But, OCaml is also used in bioinformatics [21–23], structural bioinformatics [24–27], chemoinformatics [14, 28], systems biology [29–32] and ecotoxicology [33].

There are several industrial users of the language [34] including Bloomberg, Citrix, Dassault Systèmes, Facebook [35], Jane Street (a proprietary high frequency trading firm) and Microsoft.

OCaml has some successes in the industrial world: Lexifi’s Modeling Language for Finance [36], the ASTRÉE Static Analyzer [37] used by Airbus to certify on-board software and Microsoft’s static driver verifier [38]. OCaml has several successes in the open-source world too: the Unison file synchronizer [39], the MLdonkey [40] multi-protocol peer-to-peer client, the Coq [17] proof assistant [41] and FFTW’s symbolic optimizer of fast Fourier transforms [42].

In the remaining of this article, resources to learn OCaml are listed in “Resources to learn OCaml” section. Explanations on types and how to read signatures of OCaml functions are given in “Understanding OCaml type signatures” section. Tools for proficiency in OCaml are listed in “An OCaml programming environment” section. Several uses cases of OCaml in Chemoinformatics and Structural Bioinformatics are given in “OCaml in chemoinformatics and structural bioinformatics” section. The parallelization of scientific programs is dealt with in “Accelerating chemoinformatics and structural bioinformatics in OCaml” section. Finally, strengths and weaknesses of the language and ecosystem are discussed (“Scientific software prototyping in OCaml” and “OCaml language and ecosystem drawbacks” sections), before concluding.

## Methods

### Resources to learn OCaml

There are several books introducing the language [43–45], some of them freely available online [46–48]. Other books [49, 50] give an excellent introduction to functional programming.

The “Caml Trading” video, a talk given at Carnegie Mellon university [51], explains in details why OCaml was chosen by a high frequency trading firm [52, 53]. Like researchers, this company has the technical requirements of correctness, agility and performance.

To give a try at the language within a browser, OCamlPRO offers an OCaml interpreter and some basic lessons [54]. To learn the language via the official documentation online [55], here are the essential chapters: Chapter 1 “The core language”, Chapter 2 “The module system”, Chapter 4 “Labels and variants”, The Pervasives module (a set of functions which is always available to the programmer), The list module (the most useful data structure in functional programming). One should be able to start programming in OCaml after having read only this material.

The standard library documentation is available online [56]. While it allows one to have an idea of the standard modules and their capabilities, it is not recommended for large scale software development. For real world programming, an extended standard library is necessary. For example OCaml-containers (code [57] and documentation [58]) or OCaml batteries-included (code [59] and documentation [60]) or Janestreet’s core (code [61] and documentation [62]).

**Fig. 1:**
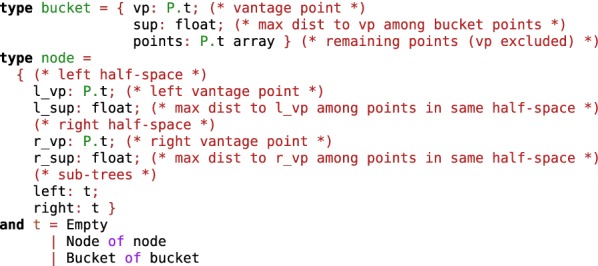
OCaml code defining a bucketized bisector-tree. The code is parameterized by a point type (P.t). The implementation works with any point type, as long as it defines a distance function

To give a taste of OCaml, Fig. 1 shows the complete definition of a bisector-tree [63]. A bisector tree is a data structure to store n-dimensional points provided a distance function between those points exists. Such a tree allows to do fast nearest neighbor searches and orthogonal queries [64]. Vantage point trees [65, 66] and $\mu$-trees [67] are closely-related data-structures which could be used for the same purpose. Our implementation (opam package bst [68]) is parameterized by a distance function and bucketized, i.e. leaves of a tree can hold up to $k \ge 1$ (user-chosen parameter) molecules.

### Understanding OCaml type signatures

A type signature is a formal specification of the behavior of a function. Unfortunately, most of the time, this specification is incomplete and unless the function’s name is explicit enough, reading the documentation is necessary to understand the complete specification.

Since being able to read type signatures is essential in OCaml, we list in code as well as in plain English some of the type signatures of essential functions of the list module. The list module uses polymorphic types, i.e. a list can contain elements of any type, but a given list can only contain elements of the same type. $\alpha$ and $\beta$ are standard names for polymorphic types.

For brevity later on, a few definitions are given hereafter.

#### **Definition 1**

The syntax

$$\texttt {apply} :\alpha \rightarrow \beta$$

defines the type of a function named apply from type $\alpha$ to type $\beta$ in which $\alpha$ and $\beta$ are type parameters. The equivalent C++ header file portion would be 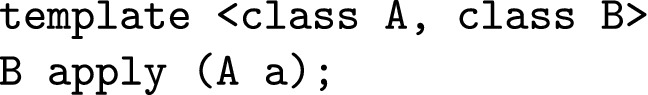


#### **Definition 2**

Let’s call accumulate any function which takes an $\alpha$, a $\beta$ and returns an $\alpha$.

$$\texttt {accumulate} :\alpha \rightarrow \beta \rightarrow \alpha$$

#### **Definition 3**

Let’s call side-effect any function which takes an $\alpha$ and returns nothing (in OCaml, nothing’s type is called unit).

$$\texttt {side-effect} :\alpha \rightarrow unit$$

#### **Definition 4**

Let’s call predicate any function which takes an $\alpha$ and returns a Boolean.

$$\texttt {predicate} :\alpha \rightarrow bool$$

#### **Definition 5**

Let’s call comparison any function which takes two alphas and returns an integer.

$$\texttt {comparison} :\alpha \rightarrow \alpha \rightarrow int$$

Then, it becomes possible to explain some list functions and their type signatures.

cons: $$\alpha \rightarrow \alpha \ list \rightarrow \alpha \ list$$
  The cons (construct) function takes an $\alpha$, a list of alphas and returns a list of alphas. The :: syntax operator is also available for the cons function. Hence, the OCaml expression $\texttt {1 {:}:}$ [2;3;4] constructs the list [1;2;3;4] and $\alpha = int$.
hd: $$\alpha \ list \rightarrow \alpha$$
  hd (head) takes a list of alphas and returns an $\alpha$ (the first one in the list).
tl: $$\alpha \ list \rightarrow \alpha \ list$$
  tl (tail) takes a list of alphas and returns a list of alphas (all elements of the list except the first one). Note that head and tail will raise an exception if called on the empty list [].
length: $$\alpha \ list \rightarrow int$$
  length takes a list of alphas and returns an integer.
map: $$(\alpha \rightarrow \beta ) \rightarrow \alpha \ list \rightarrow \beta \ list$$
  This is the map function in Google’s map-reduce [69]. map takes an apply, a list of alphas and returns a list of betas. Using the function with $\alpha = \beta$ is possible, but having the type signature using $\alpha$ and $\beta$ makes the function more generic.
fold: $$(\alpha \rightarrow \beta \rightarrow \alpha ) \rightarrow \alpha \rightarrow \beta \ list \rightarrow \alpha$$
  The reduce in Google’s map-reduce [69] is a kind of fold. fold takes an accumulator, an $\alpha$, a list of betas and returns an $\alpha$.
iter: $$(\alpha \rightarrow unit) \rightarrow \alpha \ list \rightarrow unit$$
  iter (iterate) takes a side-effect, a list of alphas and returns nothing.
exists: $$(\alpha \rightarrow bool) \rightarrow \alpha \ list \rightarrow bool$$
  exists takes a predicate, a list of alphas and returns a Boolean.
filter: $$(\alpha \rightarrow bool) \rightarrow \alpha \ list \rightarrow \alpha \ list$$
  filter takes a predicate, a list of alphas and returns a list of alphas (the ones satisfying the predicate).
partition: $$(\alpha \rightarrow bool) \rightarrow \alpha \ list \rightarrow \alpha \ list * \alpha \ list$$
  partition takes a predicate, a list of alphas and returns a pair of list of alphas (elements satisfying the predicate on the left, others on the right).
sort: $$(\alpha \rightarrow \alpha \rightarrow int) \rightarrow \alpha \ list \rightarrow \alpha \ list$$
  sort takes a comparison, a list of alphas and returns a list of alphas (sorted according to the order defined by the comparison function).

Programming most parts of the list module from scratch is an excellent exercise for any student of the language.

### An OCaml programming environment

Here follows a selection of tools for OCaml programming in a UNIX-like environment. While different users may use different tools, some of them are quite standard in a productive and modern development environment.

**Fig. 2:**
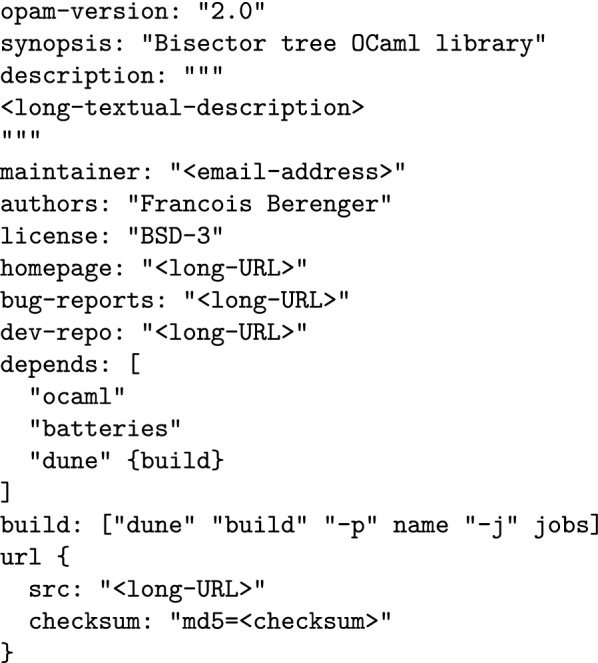
OPAM package description file for the bisector tree library. Such a file allows OPAM to automatically install/uninstall from source this library and all its transitive dependencies

**Fig. 3:**
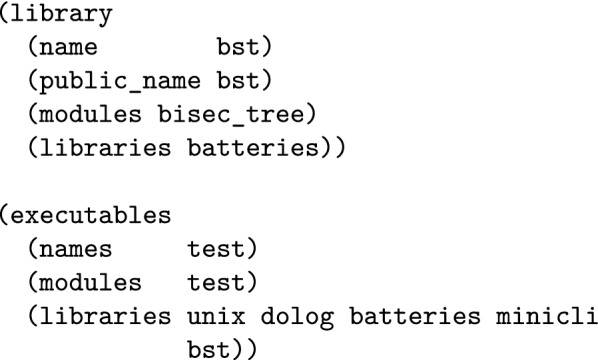
Complete build description file for the bisector tree library and it’s test executable

OPAM: the OCaml Package Manager [70] allows to automatically install OCaml software, libraries (Fig. 2) and their dependencies (even system ones). OPAM is a source-based, user-level package manager. It can install a given compiler version and packages in a so-called “switch”, under the user’s home directory. The collection of open source OPAM packages is maintained by the community [71].
opam-bundle: can create a stand-alone, self-extracting and automatic installer for any OCaml software with an OPAM package description file [72].
utop: utop [73] is an improved top-level (interactive interpreter). Utop supports line editing, history, automatic completion, colorful syntax highlighting and more. Utop can be controlled within Emacs or as a standalone terminal application. In the Python world, the equivalent of utop would be ipython.
Merlin: is an editor helper [74]. It provides completion, type information and source browsing (jump to definition/list uses) for Vim and Emacs. Thanks to Merlin, standard editors become full integrated development environments for OCaml.
Emacs: with modes like tuareg, ocaml or merlin, writing OCaml programs under Emacs is productive. Vim also has good support for OCaml. Microsoft Visual Studio Code [75] and Atom [76] also have some support for OCaml.
Dune: is the best choice to manage the compilation of OCaml projects. It is very fast, has no system dependencies and supports parallel builds on all platforms. Build descriptions are terse but still human-readable (see Fig. 3).
ocp-browser: is a terminal program to browse the interface and documentation of all installed OCaml libraries in an OPAM switch. ocp-browser alleviates the need to search and read HTML documentation online while programming.
ocp-indent & ocamlformat: automate and standardize the indentation of OCaml source code. ocp-indent [77] and ocamlformat [78] integrate well with Emacs and Vim.

## Results

### OCaml in chemoinformatics and structural bioinformatics

We list some open source OCaml software that resulted from research in chemoinformatics and structural bioinformatics [79].

**Fig. 4:**
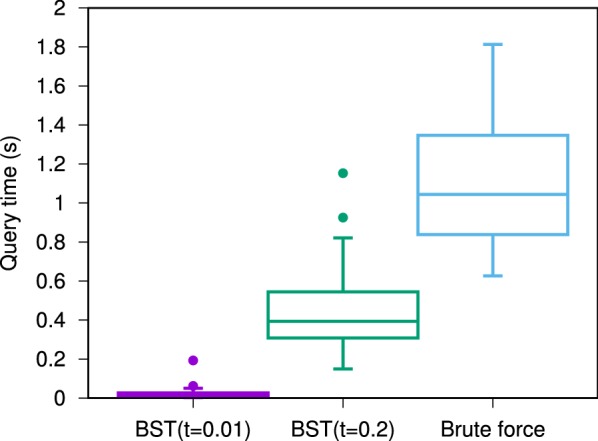
Using a bisector-tree (BST) to accelerate similarity searches on a database of $10^6$ PUBCHEM molecules. Molecular encoding is Faulon’s signature molecular descriptor [80] (an unfolded-counted fingerprint) with height equal to one bond and parametrized over MOL2 atom types. The database is searched for all molecules with Tanimoto to query $\ge 0.99$ (left; t = 0.01) or Tanimoto $\ge 0.8$ (middle; t = 0.2). The brute force version is shown on the right. 50 molecules from the database were selected randomly to serve as queries. Creating the BST (database indexing) took approximately 5 min using a single core of our desktop computer

The bisector-tree data-structure describbed in the introduction is not a toy example. It can be used to accelerate similarity searches (Fig. 4).

For ligand-based virtual screening in 3D, the AutoCorrelation of Partial Charges method (ACPC [28]) uses the autocorrelation function [81] and linear binning [82] to encode all atoms of a molecule into a rotation-translation invariant representation. ACPC allows to rank-order a database of compounds versus a query molecule and was released in open source (opam package acpc [83]). ACPC performed remarkably well in retrospective ligand-based virtual screening experiments. At an average speed of 1649 molecule/s, ACPC reached an average median area under the curve of 0.81 on 40 Directory of Useful Decoys [84] targets.

Consent [14, 85] (opam package lbvs_consent [86]) performs ligand-based virtual screening using consensus queries. When several active molecules are known, screening with all of them is recommended (instead of using just one). A consensus query can be created by screening serially with different ligands before merging similarity scores, or by combining chemical fingerprints. Consent was tested on 19 protein targets, 3776 known active and $\sim 2\times 10^6$ inactive molecules from high throughput screening datasets. Three fingerprints were investigated (MACCS, ECFP4 and an unfolded fingerprint). Different consensus policies and consensus sizes (number of known actives) were benchmarked. A consensus fingerprint is always faster. In some circumstances, it can approach the performance of a consensus of scores in terms of Area Under the Receiver Operating Characteristic (ROC) Curve (AUC) and early retrieval.

**Fig. 5:**
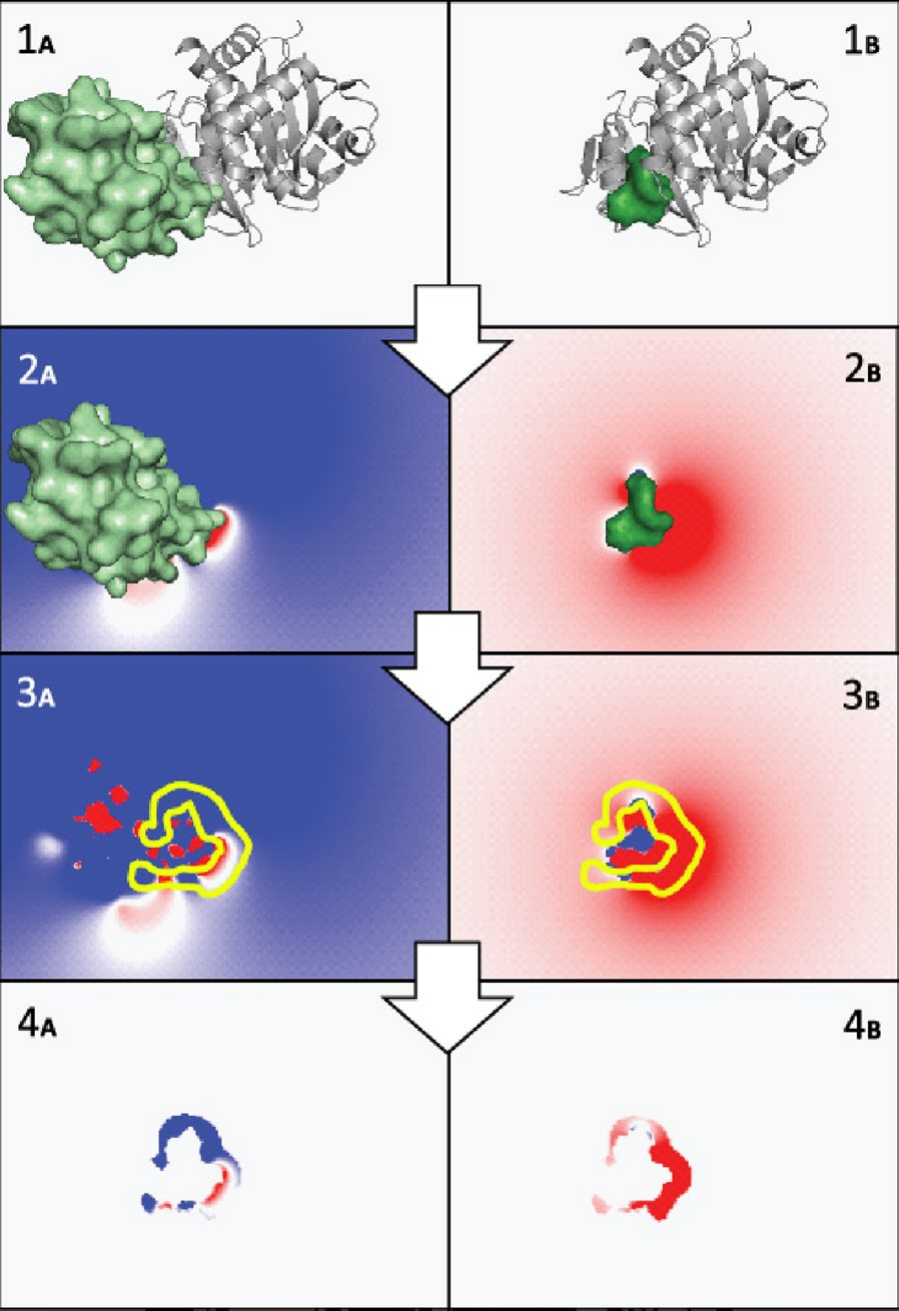
Overview of EleKit applied to PDB codes 2B4J ($1_{A}$) and 3LPU ($1_{B}$). The “ligand-protein” is shown as a green surface in $1_{A}$ and $2_{A}$. The “ligand-small-molecule” is shown as a smaller green surface in $1_{B}$ and $2_{B}$. The receptor protein is shown as a gray cartoon in $1_{A}$ and $1_{B}$. Electrostatic potential fields are calculated and stored in distinct grids ($2_{A}$ and $2_{B}$). A boolean mask in 3D is created to select the solvent region nearby the interface ($3_{A}$ and $3_{B}$). Finally, the similarity between electrostatic potentials in the masked region ($4_{A}$ and $4_{B}$) is calculated using the Spearman rank correlation coefficient (figure adapted from Voet [26])

EleKit [26, 27] was the first structural bioinformatics software able to measure the similarity of a ligand’s electrostatic field with that of a protein binding at a protein-protein interface (Fig. 5). Ligands showing a high similarity in this setting are potential drugs breaking protein-protein interactions. EleKit was a complex software, driving PDB2PQR [87], parsing PQR files, running the Adaptive Poisson-Boltzmann Solver (APBS [88]) in parallel, parsing ABPS output files, creating and operating 3D Boolean masks.

Also in structural bioinformatics, Fragger [25, 89, 90] is a protein fragment picker for 3D structural queries. From a set of PDB files, Fragger can create a protein fragments database. All fragment lengths are supported. Using the triangular inequality, Fragger can efficiently search with a query fragment and a distance threshold. Matching fragments are ranked by distance to the query, which can contain structural gaps. The allowed amino acid sequences matching a query can be constrained. Fragger is meant for protein design, loop grafting and related activities.

### Accelerating chemoinformatics and structural bioinformatics in OCaml

**Fig. 6:**
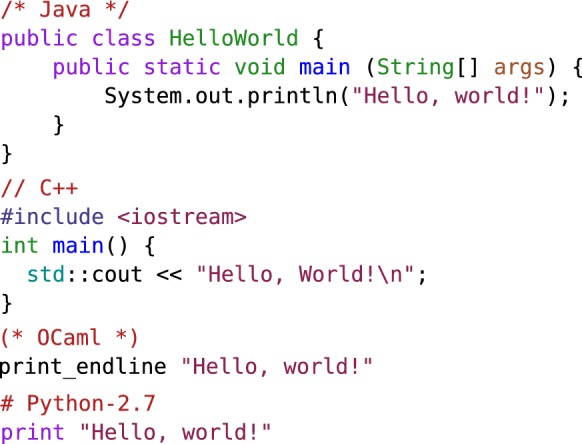
Valid hello world programs to illustrate the *idiomatic* verbosity of Java, C++, OCaml and Python-2.7. Keep in mind that in many programming languages, programmers can make their source code arbitrarily small, sometimes to the point that a program is no more readable

OCaml executables are fast. In terms of speed, OCaml is placed just after Go in the Debian language shootout [91]; the fastest language being C++ then C. However, execution speed is not the most important in a research setting. Programmer productivity is more important. In terms of verbosity, OCaml code is close to Python and far from Java (see Fig. 6). From past experience, an AUC calculation in OCaml is about 20 times faster than the equivalent python script [92]. While performing an AUC calculation faster may not seem important, to scientifically validate a computational method, one might run thousands of such calculations.

**Fig. 7:**
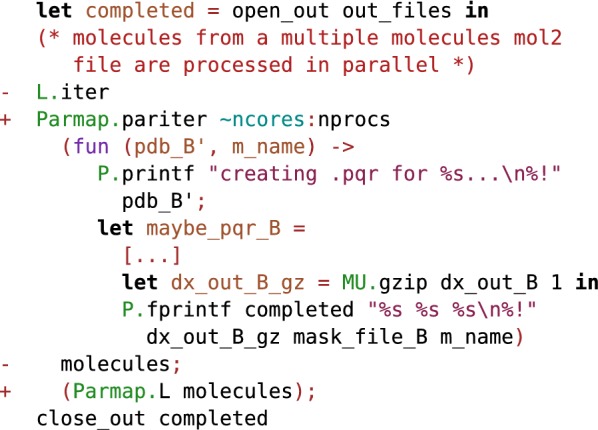
Git diff after parallelization of EleKit. Parallelizing EleKit using Parmap was a two lines change in $\sim 3000$ lines of code. All program development and debugging was done on sequential code. With an electrostatic calculation run-time of approximately 2 min per small molecule, parallelization was mandatory for production use of EleKit on thousands of molecules

**Fig. 8:**
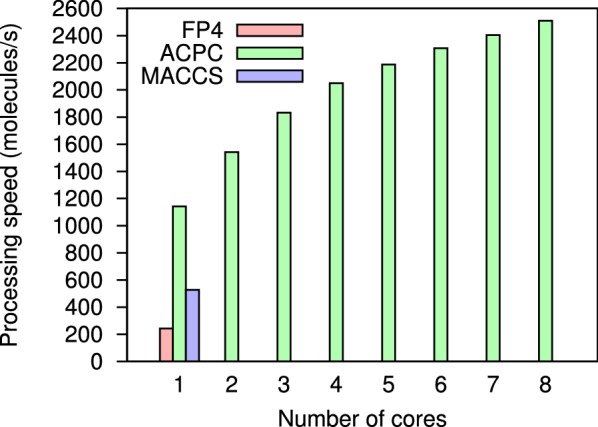
Performance of ACPC in the electrostatic space, using Parmap for parallelization. Open Babel 2.3.9’s MACCS and FP4 C++ implementations run-times are shown to give an order of magnitude. Run-times were averaged over three runs. Protein target: Human immunodeficiency virus type 1 protease (HIVPR) from the Database of Useful Decoys Enhanced (DUDE [93]); 26450 ligands and decoys

**Fig. 9:**
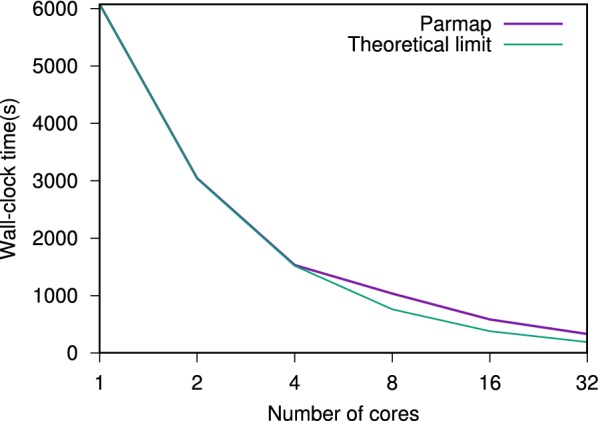
Wall-clock time to analyze hundreds of molecules with EleKit and Parmap. Up to four cores, the parallelization performance is almost indistinguishable from a perfectly parallelizing program (theoretical limit)

Since molecules can be processed independently, most chemoinformatics tasks are easy to parallelize. The Parmap OCaml library [94] provides parallel iter, map and fold functions for arrays and lists on multi-core computers. Parallelizing code with Parmap is trivial (Fig. 7). Parmap preserves semantics while achieving nearly optimal speedup [94] (Figs. 8, 9).

For stream computing, when a program cannot hold all items in memory (which is required by Parmap), we developed the parany library (opam package parany [95]). Parany is more generic than parmap. It is structured around three functions. An unfold function called demux, an apply function called work and a fold/reduce function called mux.

- $$\texttt {demux} :unit \rightarrow \alpha$$
- $$\texttt {work} :\alpha \rightarrow \beta$$
- $$\texttt {mux} :\beta \rightarrow unit$$

Some more complex technologies exist to write even higher performance OCaml programs. SPOC [96] is an OCaml library allowing general purpose GPU programming, using Cuda or OpenCL kernels. SPOC allows to create specific data sets usable by those kernels and automatically manages memory transfers between CPU and GPU.

BER MetaOCaml [97] is an OCaml dialect for multi-stage programming [98]. It allows run-time C code generation and program execution. BER MetaOCaml can be used to compile domain-specific languages and automate the specialization of high-performance computational kernels.

## Discussion

### Scientific software prototyping in OCaml

**Fig. 10:**
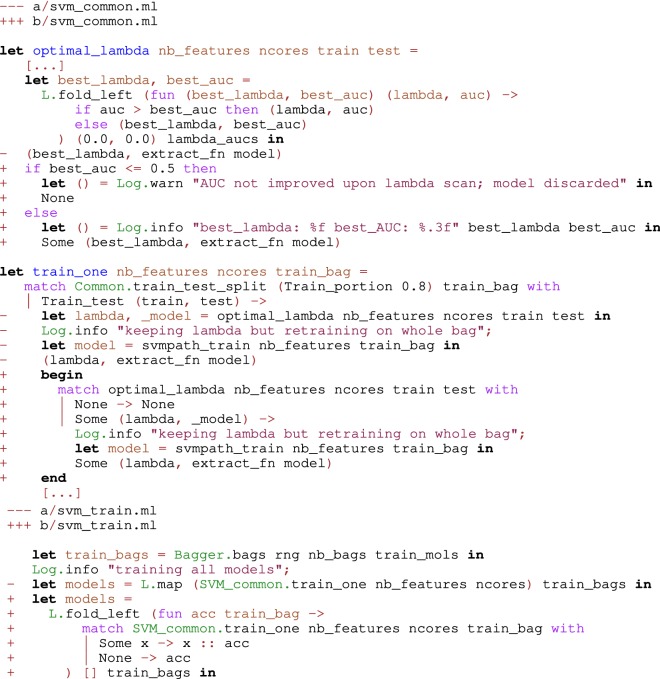
Git diff excerpt of an actual code refactoring in the SVM part of a category-QSAR software. Sometimes, the R svmpath package encounters numerical problems, like an exactly or computationally singular matrix. To deal with such rare cases, it was decided to drop a model from the bag of models. Since a bagging classifier with 21 models was being trained, dropping one or two models was deemed better than letting the whole software crash. Hence, an option type was introduced in the function optimal_lambda from file ‘svm_common.ml’, along with proper warning messages. Then, the compiler forced updating the rest of the code

In an academic research setting, it is common for a software project to be severely under staffed, compared to industrial standards, i.e. a single person might be in charge of the full software life-cycle (requirements gathering, specification, design, implementation, speed optimization, parameter tuning, test and validation, release and packaging, maintenance). In research, requirements are ill-defined and changing. Since the purpose of the software is to scientifically show that an idea works, having a high confidence in the software is important. Moreover, during the course of the project, design decisions might change and impact the whole code-base. OCaml types and compiler allow to refactor software fast and without missing any place that needs changing (see Fig. 10 for an example of refactoring that only took a few minutes). Thus, during prototyping, the programmer is not afraid to do drastic changes to the software (agility). In such a setting, and when using OCaml, we propose to abandon the practice of unit tests. Because, there is not enough manpower to write and maintain them. Note however that OCaml has tools for programmers who want to write unit [99] or property-based tests [100] as comments inside their code. Since the software will change a lot during its lifetime, maintaining unit tests would be too costly and slow down the pace of research. Of course, if we were using a dynamically-typed language such as Python or Ruby, such a decision would be risky and many problems discovered at run-time. Instead of unit tests, we propose to use regression tests and end-to-end validation, once a prototype is advanced enough. For example, a valid output can be verified by hand from a known input and added to a set of regression tests.

In the same vain, we propose to abandon OCaml interface files when prototyping. Having to maintain interface files slows down refactoring. Interface files of libraries should only be added once a project is going to be released.

When programming in OCaml, one strongly relies on the compiler to catch errors. It is common to see a complex but compiling OCaml program run without any run-time error, even when running for the first time. Programs written in Haskell have this exact same property.

### OCaml language and ecosystem drawbacks

When working in OCaml, if functors and module signatures are heavily used, compiler error messages can become hard to understand. Also, the required syntax is nontrivial and might need some practice.

For chemoinformatics, a parser for Simplified Molecular-Input Line-Entry System (SMILES [101]) and a parser for SMiles ARbitrary Target Specification (SMARTS [102]) are the most obvious missing libraries. Also, nowadays it would not be reasonable to do chemoinformatics research without using the functionalities of the Chemistry Development Kit (CDK [103, 104]), Rdkit [105] or Open Babel [106]. Since there are no OCaml bindings to those libraries, our current solution is to write small programs interfacing with them, in order to extract or import data to/from them. By following the UNIX design principles [107], it is easy to create, debug and maintain software that exchange data via text files. However, in some projects [14, 26, 28], we have written parsers for parts of the PDB [108], PQR [87] and MOL2 [109] file formats.

Currently, the OCaml ecosystem is weak in the Machine Learning field, especially when compared to Python and the Scikit-learn [110] library. At least, there is one library for classification using random forests [111] (opam package orandforest [112]) and a numerical library (opam package owl [113, 114]) with some machine learning functionalities like regression and neural networks. For deep learning, some OCaml bindings to TensorFlow [115, 116] and PyTorch [117] have been released recently. To palliate the deficiency in machine learning libraries, we have recently developed several OCaml packages taping into the R [118] ecosystem; for support vector machines (opam package orsvm-e1071 [119]), random forests (opam package orrandomForest [120]) and gradient boosted trees (opam package orxgboost [102]). We have also developed the classification performance metrics library in order to benchmark virtual screening experiments (opam package cpmlib [121]). Cpmlib features ROC curves, AUC [122], enrichment factor, power metric [123] and Boltzmann-Enhanced Discrimination of ROC (BEDROC [124]).

**Fig. 11:**
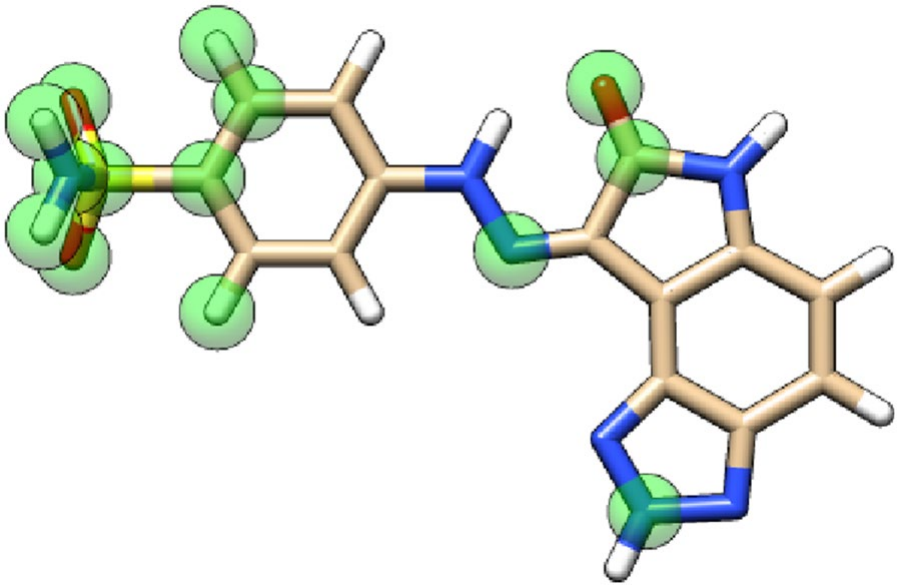
Graphical annotation of a query molecule using a BILD file generated by the ACPC software for viewing with UCSF Chimera. A query molecule of the CDK2 protein target is annotated in the electrostatic space, based on atomic contributions to AUC. Transparent green balls highlight atoms which if masked (their contribution is removed from the molecular encoding/fingerprint) would decrease the AUC reached by this molecule in a similarity search

OCaml is best for back-end and system [125] programming. To quickly annotate molecules or protein structures, rather than doing graphics programming in OCaml, we recommend generating BILD [126] files. BILD files are simple, human-readable line-oriented text files, easy to generate by a program or by hand. They can be viewed within UCSF Chimera [127] (Fig. 11).

While OCaml is a portable language, not all programmers write portable programs. OCaml code can be automatically translated to JavaScript [128] to target web browsers (opam package js_of_ocaml). But parallel programs or programs relying extensively on the Unix module might not work under Windows. Also, there may be less libraries/opam packages available under Windows. If Windows support is a primary concern, F# or Haskell [16] might be safer programming language choices. If access to a comprehensive chemoinformatics library is a prime concern, Scala might be a safer choice since its interoperability with Java would allow using the Chemistry Development Kit.

For managers, the fact that there are few OCaml programmers available on the market is a concern. However, we feel that programmers can become proficient in the language quickly, so this is not a major concern.

## Conclusions

OCaml is a strongly typed programming language of the functional family. In this article, we have tried to share our experience in using it for Chemoinformatics and Structural Bioinformatics research.

This article should not be seen as an attempt at asserting the superiority of OCaml and/or functional programming over other programming languages and approaches. Rather, we encourage researchers to choose and use the tools that make them the most productive, even if those tools are not mainstream.

To us, OCaml has been proven quite productive for software prototyping in Chemoinformatics and Structural Bioinformatics method development. The software demonstrated here were used intensively and timely during scientific validation campaigns, on many molecules and protein targets. We have never regretted our choice of OCaml and still use it today.
